# Expression of concern

**DOI:** 10.1530/EJE-20-0857x

**Published:** 2021-02-01

The Editor-in-Chief and Editorial Board of the *European Journal of Endocrinology* (EJE) are publishing an Expression of Concern regarding a recently published article in the journal. The Expression of Concern is to alert readers that an active investigation is underway into an allegation made regarding this article and the data reported within it:

Ye X, Zhao H, Liu J, Lu B, Shao J & Wang J. Efficacy and safety of tripterygium glycosides for active moderate to severe Graves’ ophthalmopathy: a randomised, observer-masked, single-centre trial. *European Journal of Endocrinology* 2021 **184** 277–287. (https://doi.org/10.1530/EJE-20-0857)

The paper will not be published in print until the investigation has been completed.

An update to this notice will be published online when the investigation is concluded.

